# YOLOv8-PD: an improved road damage detection algorithm based on YOLOv8n model

**DOI:** 10.1038/s41598-024-62933-z

**Published:** 2024-05-27

**Authors:** Jiayi Zeng, Han Zhong

**Affiliations:** College of information and Network Safety, People’s Public Security University of China, Beijing, 100038 China

**Keywords:** Pavement distresses, YOLOv8-PD, Attention mechanism, GhostNet, LSCD-Head, Computer science, Information technology

## Abstract

Road damage detection is an crucial task to ensure road safety. To tackle the issues of poor performance on multi-scale pavement distresses and high costs in detection task, this paper presents an improved lightweight road damage detection algorithm based on YOLOv8n, named YOLOv8-PD (pavement distress). Firstly, a BOT module that can extract global information of road damage images is proposed to adapt to the large-span features of crack objects. Secondly, the introduction of the large separable kernel attention (LKSA) mechanism enhances the detection accuracy of the algorithm. Then, a C2fGhost block is constructed in the neck network to strengthen the feature extraction of complex road damages while reducing the computational load. Furthermore, we introduced lightweight shared convolution detection head (LSCD-Head) to improve feature expressiveness and reduce the number of parameters. Finally, extensive experiments on the RDD2022 dataset yield a model with parametric and computational quantities of 2.3M and 6.1 GFLOPs, which are only 74.1% and 74.3% of the baseline, and the mAP reaches an improvement of 1.4 percentage points from the baseline. In addition, experimental results on the RoadDamage dataset show that the mAP increased by 4.2% and this algorithm has good robustness. This method can provide a reference for the automatic detection method of pavement distress.

## Introduction

Road networks are the foundation of economic development^1^, a good transport system can meet people ’s travel needs, business and industrial development. However, these highways face challenges from infrastructure deterioration. To maintain the momentum of national economic development, it is necessary to protect transportation infrastructure. Pavement cracks are common road damages, which can affect road safety and driving safety. Maintaining high-quality pavements is one of the keys to ensuring road safety, and road damage detection can play an important role in preventing road damage and maintaining traffic road safety^2^. Traditional manual inspections are costly in terms of time, with long monitoring periods and low efficiency. Therefore, the development of efficient and lightweight technology for pavement distress detection is of great significance.

Road damage target detection technology is mainly divided into traditional target detection algorithms and target detection algorithms based on deep learning. Traditional crack detection methods mostly rely on manual inspection^3–5^ or image segmentation techniques that recognize pavement distress by extracting features. It is difficult for artificial methods to extract features effectively when the road environment changes. Because of that, the robustness of these algorithms is poor and the detect process is tedious^6^.

In recent years, with the rapid development of deep learning technology, deep learning has gained widespread attention in many fields^7–10^, as well as target detection. Deep learning networks offer exceptional speed and accuracy in target detection tasks, demonstrating strong robustness and generalization capabilities. By circumventing the need for manual feature extraction and intricate feature segmentation operations, deep learning minimizes the risk of misclassifying or omitting crucial target features during pre-feature sampling^11^. In order to solve the bottleneck of cell instance segmentation and tracking based on cosine embedding, Zhao et al.^12^ proposed a faster mean shift algorithm. This algorithm provides a plug-and-play model suitable for cluster reasoning based on pixel embedding and makes the road defect detection based on neural network can be widely used.

Target detection technology is mainly divided into single-stage algorithms and two-stage algorithms. Typical examples of two-stage algorithms are R-CNN^13^, Fast R-CNN^14^, Faster R-CNN^15^ and SPP-Net^16^. Kang D et al.^17^ used an ensemble of the Faster R-CNN algorithm to detect crack regions. Haciefendio et al.^18^ used Faster R-CNN to detect defects in concrete pavements. Pei et al.^19^ used the Cascade R-CNN model and various data augmentation techniques, achieving an F1 score of 0.635 in global road damage detection. Yamaguchi et al.^20^ developed a method for accurately assessing road cracks using U-Net through LiDAR data enhancement and morphological transformation. Arya et al.^21^ used the lightweight network MobileNet to detect road damage images from the RDD2020 datasets, achieving an F1 score of 0.52. Zhong et al.^53^ proposed a multi-scale feature fusion deep neural network structure w-SegNet based on the SegNet network, which has strong robustness for crack detection in various scenarios. The aforementioned studies has made contributions to road damage detection, but there is still a lot of room for improvement in accuracy and detection speed.

Single-stage object detection algorithms include the You Only Look Once (YOLO) series^22,23^, Single Shot MultiBox Detector (SSD)^24^, and Retinanet^25^, etc. Although single-stage algorithms have slightly lower detection accuracy compared to two-stage algorithms, they excel in detection speed. Therefore, single-stage algorithms received more attention in road damage detection. Mandal et al.^26^ proposed the use of YOLO CSPDarknet53 network for road defect detection, but there is still much room for improvement in both accuracy and detection speed. Fang Wan et al.^27^ proposed a lightweight road defect detection algorithm, YOLO-LRDD, which used the novel backbone network Shuffle-ECANet to reduce the model size while maintaining accuracy. It is suitable for deploying on mobile devices. Zhang et al.^28^ described a multi-level attention mechanism, called multi-level attention block, to strengthen the utilization of essential features by the YOLOv3. Zhong et al^52^ introduces an enhanced Wasserstein Generative Adversarial Network with gradient penalty (WGAN-GP) to generate realistic 512 $\times$ 512 pixel images of grooved cement concrete pavement cracks. The model improves detection robustness, with YOLOv3 achieving a 6% higher mean average precision using the augmented dataset. Yu^29^ proposed a road crack detection algorithm based on YOLOv5 and made a lightweight improvement, which significantly reduced the size of the model parameters and improved the detection speed. YOLOv5 also proves to be a perfect fit for real-time detection^30,31^ due to its speed and considerable accuracy. Zhong et al.^32^ proposed an pavement distress detection algorithm named PDDNet which utilizes three algorithms, YOLOv4, YOLOv5, and YOLOv7, for object detection and localization in UAV images. The experimental data showed that the accuracy of this algorithm was better than R-CNN and U-Net. Roy et al.^33^ presented an DenseSPH-YOLOv5 road damage detection model by using Swin-Transformer Prediction Head that can improve efficient detection of multiscale object sizes and simultaneously reduce the computational complexity. The YOLO algorithm, as one of the classic single-stage detection algorithms, has been updated to YOLOv8, which has significant advantages in both detection accuracy and efficiency. Therefore, we choose to optimize the model based on YOLOv8, to further improve its accuracy and reduce its size.

## Methodologies

### YOLOv8n

**Figure 1:**
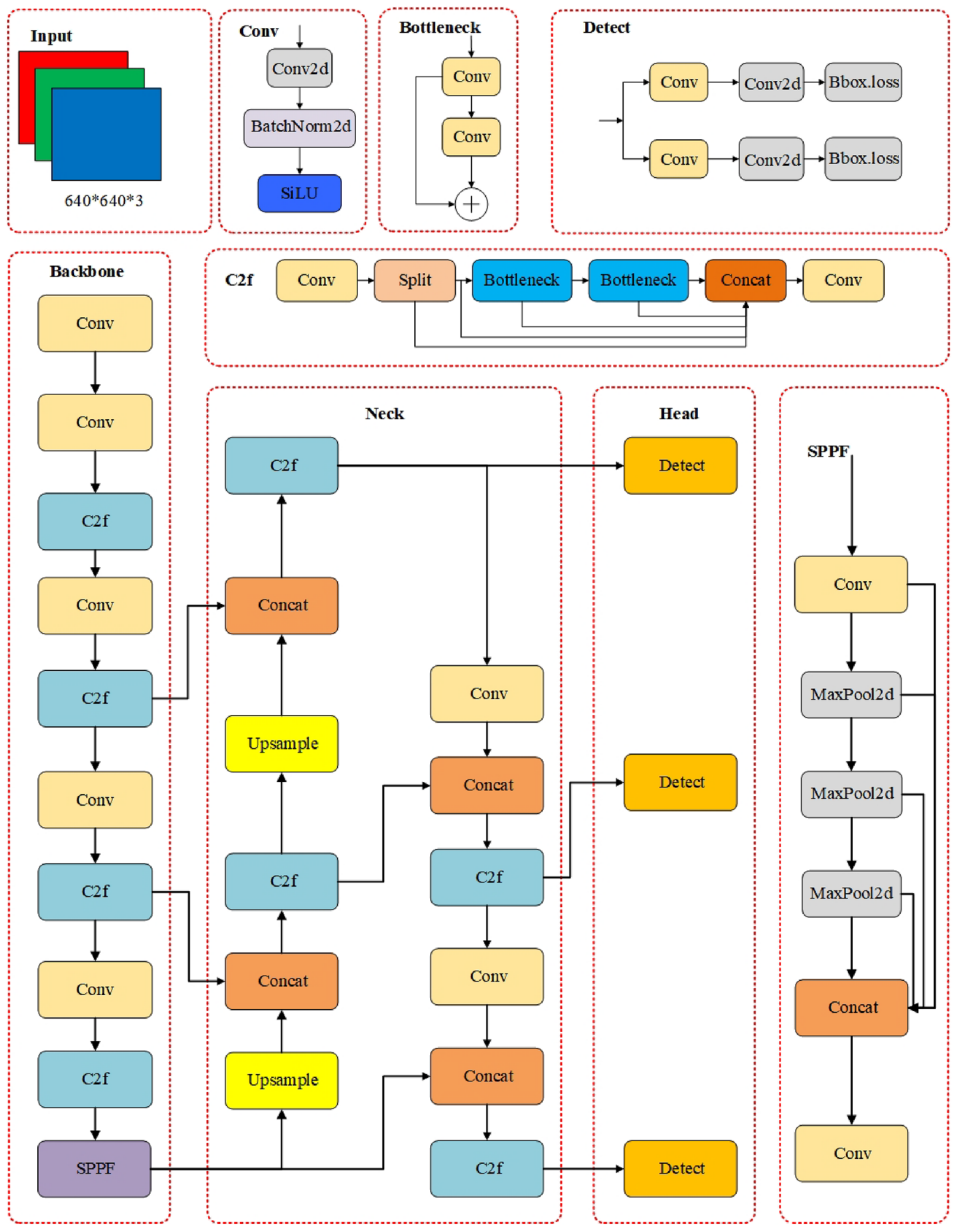
YOLOv8n network architecture.

In recent years, the YOLO algorithm has been continuously optimized and updated. In 2023, the Ultralytics team introduced the YOLOv8 model, which incorporates new features and improvements to further enhance performance and flexibility. Firstly, the new model replaced the C3 structure of YOLOv5 with the gradient-rich C2f. structure and adjusted the number of channels. The C2f.structure retains the advantages of the ELAN structure in YOLOv7^34^. This structure reduces a standard convolutional layer and uses the Bottleneck module to enhance the gradient branch. Secondly, the head section was also modified to separate classification and detection using the decoupling head technique. Furthermore, the loss function utilized positive-negative matching of samples instead of IOU matching. These improvements streamline the YOLOv8n network structure, increasing detection speed and improving detection accuracy. The Yolov8n model has proved to be the most lightweight road defect target detection model in recent years, especially suitable for deployment on resource-constrained devices such as drones and on-board devices. The overall structure of the Yolov8n detection model is shown in Fig. 1.

### YOLOv8-PD

**Figure 2:**
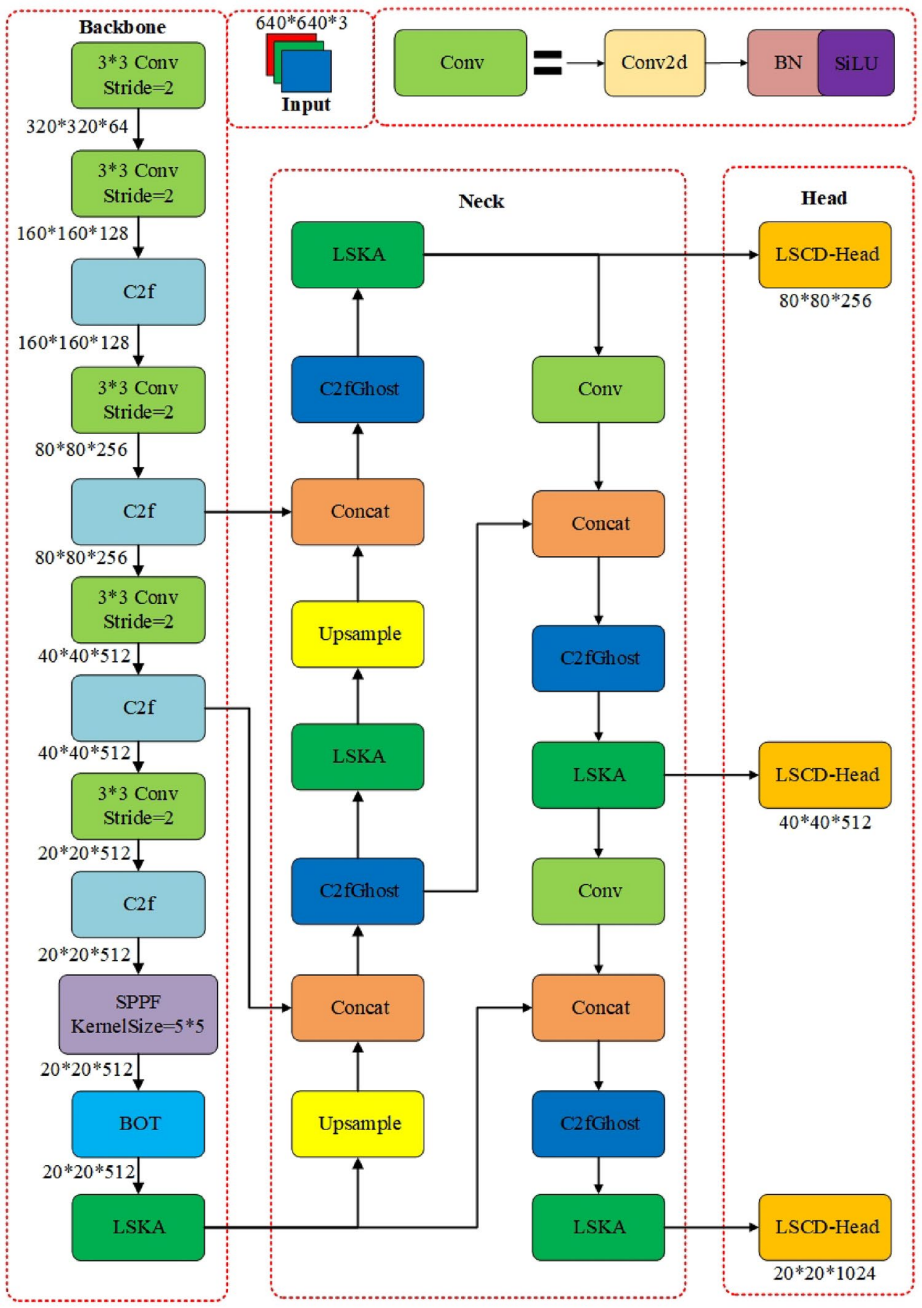
Model structure of YOLOv8-PD.

To tackle issues associated with inaccurate detection of pavement distress in conventional networks, excessive model parameters, and large model sizes, this study introduces a novel pavement distress detection model termed YOLOv8-PD (Pavement Distress) , illustrated in Fig. 2. This model can improve the detection accuracy of four pavement distresses (longitudinal cracks, transverse cracks, mesh cracks and potholes), especially for longitudinal cracks. This model has four key enhancement points. Firstly, the introduction of BOT Transformer^35^ enables better capture of long-range dependencies in road damage images, obtaining more global information. Secondly, the LSKA mechanism^36^ is introduced at the end of the backbone network and the neck network, enhancing the extraction of road defect features and improving the algorithm’s detection accuracy. Then, the C2fGhost block^37^ is constructed in the neck network of YOLOv8n, strengthening the feature extraction of complex road defects while simultaneously reducing computational load. Furthermore, a lightweight detection head module, LSCD-Head, is proposed to enhance feature expressiveness. Lastly, The loss function of this model is consistent with the YOLOv8n model which is composed of several parts, including VFL loss function in classification task and CIOU loss function combined with DFL loss function in regression task.

### BOT module

**Figure 3:**
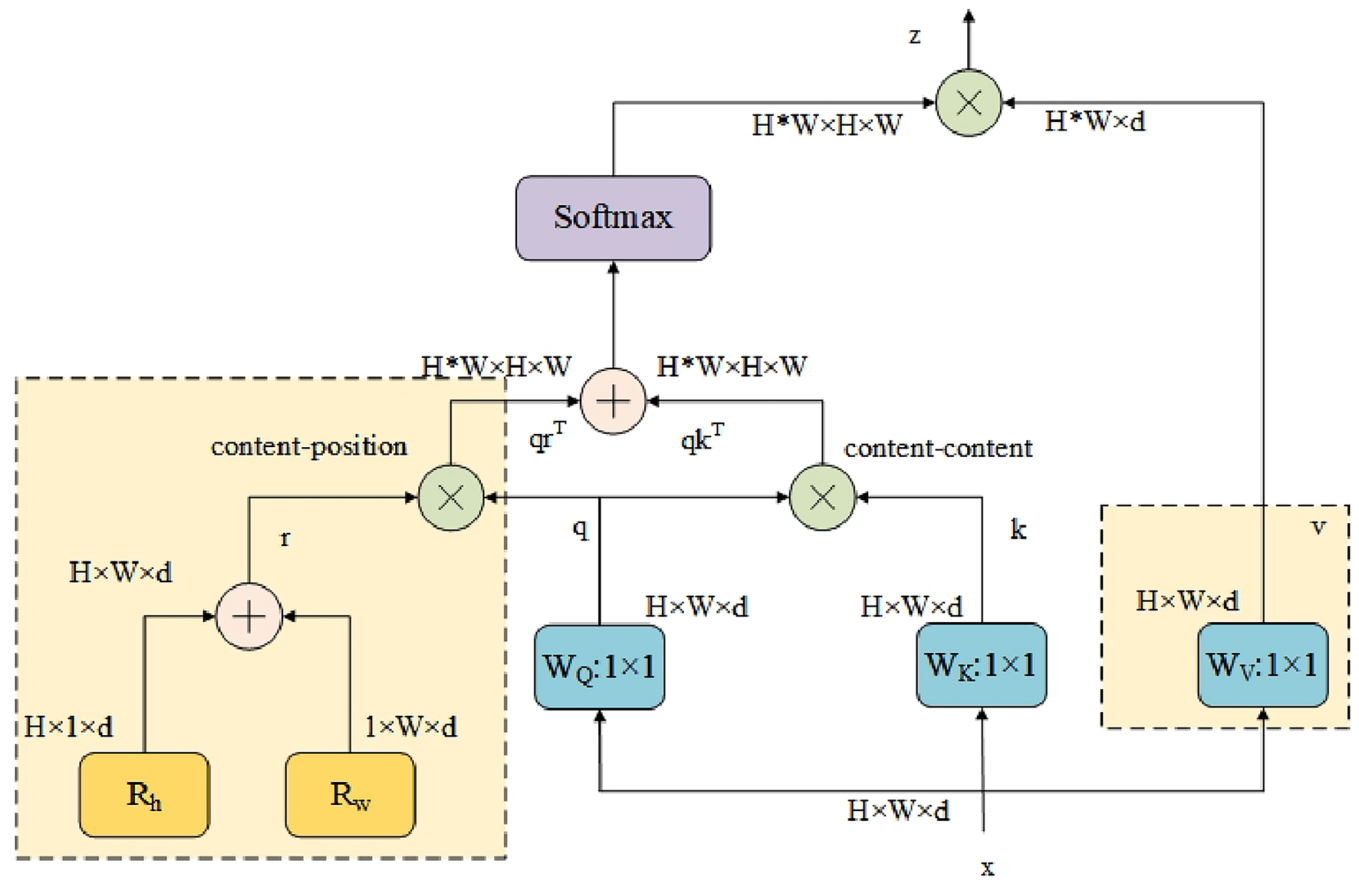
Multi head self attention layer (MHSA).

**Figure 4:**
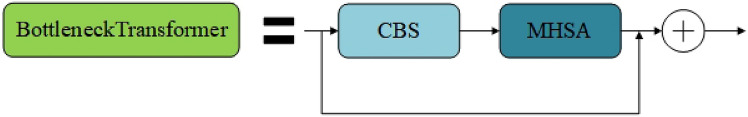
Bottleneck transformer structure.

BoTNet^35^ is a collaborative exploration by researchers from Berkeley and Google into combining convolutional networks with Transformers. It employs a hybrid approach, replacing spatial convolutional layers with multi-head self-attention (MHSA) layers from Transformers, while leveraging the feature extraction capabilities of CNNs to achieve better performance than using CNNs or Transformers alone. Figure 3 illustrates the structure of the multi-head self-attention (MHSA) layer, while Fig. 4 shows the structure of Bottleneck Transformer (BoTNet).

**Figure 5:**
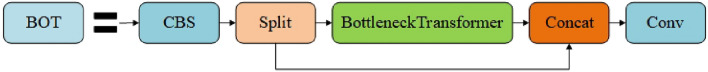
BOT module.

Most current deep learning methods for road damage detection struggle to grasp the global information of road damages. To address this issue, this paper combines the BoTNet network framework with the C2f. structure and proposes the BOT module. This module is only used in the backbone’s terminal structure, achieving a slight increase in detection accuracy with minimal computational cost. By combining the strengths of CNN and Transformer, the paper bridges the gap between them, enabling the comprehensive extraction of global and local features, thus enhancing the model’s detection accuracy. The structure of BOT module is illustrated in Fig. 5.

### Large separable kernel attention

Attention mechanisms are effective in enhancing neural representations due to their simplicity and efficiency. In the field of computer vision, many excellent attention mechanisms have been developed, including channel attention mechanisms such as SE^38^, spatial attention mechanisms such as GeNet^39^,GcNet^40^ and SGE^41^ and combined spatial and channel attention mechanisms such as CBAM^42^ and CPCA^43^ .The SKNet^44^ network introduces multiple convolution kernels to aggregate feature information along the channel dimension. Building upon SKNet, LSKA adaptively aggregates feature information from large kernels in the spatial dimension, instead of aggregating information along the channel dimension.

**Figure 6:**
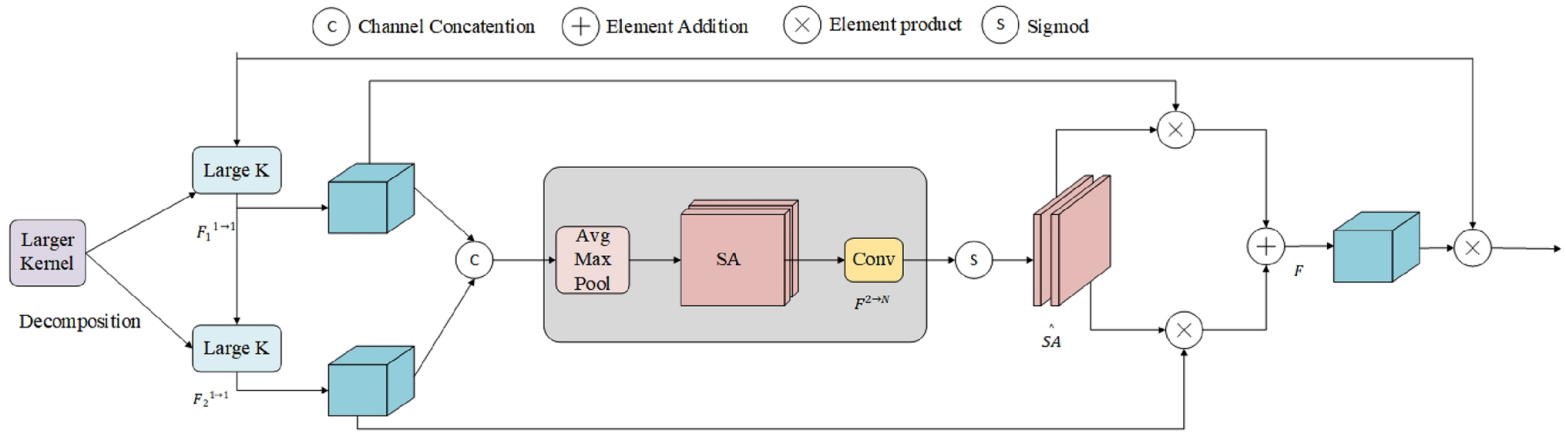
LSKA module.

Due to the complex and dynamic environment in which pavement distresses are located, in order to enhance the model’s ability to extract key crack features, LSKA decomposes large-kernel convolution operations to capture long-range dependencies and adaptability. This improves the extraction of long crack features while reducing computational complexity and memory requirements. The structure of LSKA is illustrated in Fig. 6.

To dynamically select suitable spatial kernels, the input feature map is divided into multiple sub-feature maps. Subsequently, different-sized convolutional kernels are applied to each sub-feature map, generating multiple output feature maps. These sub-output feature maps are then concatenated, as shown in Eq. (1). This concatenation leads to an increase in the channel dimension of the output feature map.

1 $$\begin{aligned} \widetilde{\textrm{U}}=\left[ \widetilde{\textrm{U}}_{1}; \ldots ;\widetilde{\textrm{U}}_{i}\right] \end{aligned}$$

Whereafter, the concatenated feature map undergoes average pooling and maxpooling operations along the channel dimension to extract spatial relationship descriptors namely $\textrm{SA}_{a v g}$ and $\textrm{SA}_{\max }$. The specific operation is illustrated in the following formulas:

2 $$\begin{aligned} \textrm{SA}_{avg}=\textrm{P}_{avg}(\widetilde{\textrm{U}}),\textrm{SA}_{\max }=\textrm{P}_{max}(\widetilde{\textrm{U}}), \end{aligned}$$

Subsequently, following the concatenation of $\textrm{SA}_{avg}$ and $\textrm{SA}_{\max }$ , convolutional layers are utilized to transform them into spatial attention maps, ensuring they possess the same number of depth convolutions N. This conversion is mathematically expressed by the following formula:

3 $$\begin{aligned} \hat{\textrm{SA}}=\textrm{F}^{2 \rightarrow N}\left( \left[ \textrm{SA}_{avg}; \textrm{SA}_{\max }\right] \right) \end{aligned}$$

By using the sigmoid activation function to each spatial attention map, the spatial selection weights for each depth convolution are obtained. The weighted depth convolution feature maps are subsequently acquired by element-wise multiplication of the weights and the corresponding depth convolutions. Finally, a convolutional layer is employed to fuse these feature maps and produce the final attention feature. This process is mathematically demonstrated through the following formulas:

4 $$\begin{aligned} \widetilde{\textrm{SA}_{i}}= & {} {\text {Sigmoid}}\left( \widehat{\textrm{SA}_{i}}\right) \end{aligned}$$

5 $$\begin{aligned} \textrm{S}= & {} \textrm{F}\left( \sum _{i=1}^{N}\left( \widetilde{\textrm{SA}}_{i} \cdot \widetilde{\textrm{U}}_{i}\right) \right) \end{aligned}$$

### GhostNet

**Figure 7:**
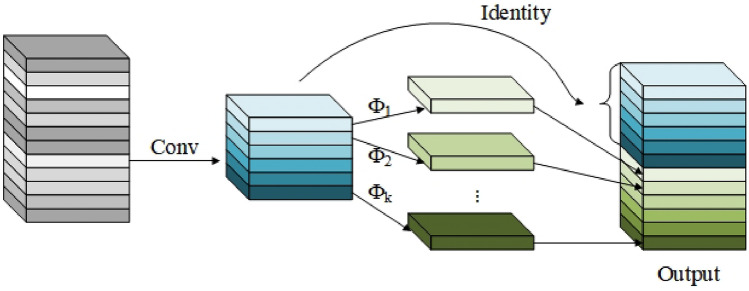
GhostNet design core.

GhostNet^37^ is a lightweight network designed by Huawei Noah’s Ark Lab in 2020. Ghostconv is a convolutional module within the GhostNet network that can replace ordinary convolutions. As shown in Fig. 7, the GhostNet network can reduce network computation and parameter volume while maintaining the channel size of the original convolution output feature map.

**Figure 8:**
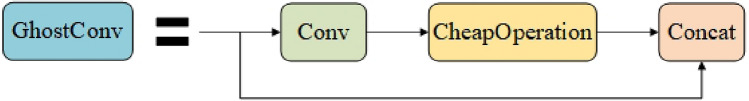
GhostConv module.

As shown in Fig. 8, the “Cheap operation” is a type of cost-effective linear operation. GhostConv first uses a convolution with half the size of the original convolution to generate half of the feature map. Then, it continues through a 5 $\times$ 5 convolution kernel with a stride of 1, performing a cost-effective calculation called “Cheap operation” to obtain the other half of the feature map. Finally, the two parts of the feature map are concatenated together through Concatenation operation to form the complete feature map.

**Figure 9:**
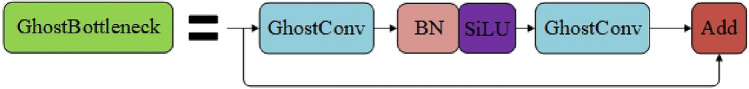
GhostBottleneck module.

As shown in Fig. 9, GhostBottleneck first passes through the first GhostConv, which acts as an expansion layer to increase the number of channels. Then, it undergoes regularization and SiLU activation function. Next, it passes through the second GhostConv to reduce the number of output feature map channels to match the input channel number. Ultimately, the feature map obtained from the previous step is added to the residual edge for feature fusion. Compared to Bottleneck, GhostBottleneck achieves higher feature extraction with fewer parameters.

**Figure 10:**
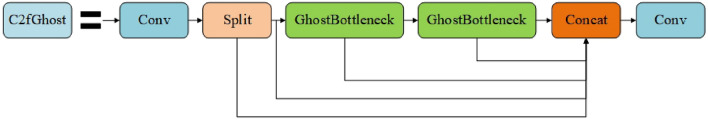
C2fGhost module.

The C2fGhost module replaces the Bottleneck layer with the GhostBottleneck layer, effectively reducing the redundant computations introduced by ordinary convolutions in Bottleneck. This reduction in parameters does not weaken the feature extraction capability. This enables the model to be deployed on mobile devices, facilitating edge computing detection of road cracks. The specific structure is illustrated in Fig. 10.

### Lightweight shared convolution detection head

The original detection head of YOLOv8 has some limitations. Firstly, the number of parameters of the detection head is large, accounting for one-fifth of the calculation amount of the entire algorithm. All three detection heads need to extract image information through two 3 $\times$ 3 convolutions and a 1 $\times$ 1 convolution, respectively. This structure results in a significant increase in the number of parameters of the algorithm. Secondly, the traditional single-scale prediction structure adopted by the original algorithm cannot deal with multi-scale targets well. It only predicts from one scale of the feature map, ignoring the contribution of other scale features to the detection.

**Figure 11:**
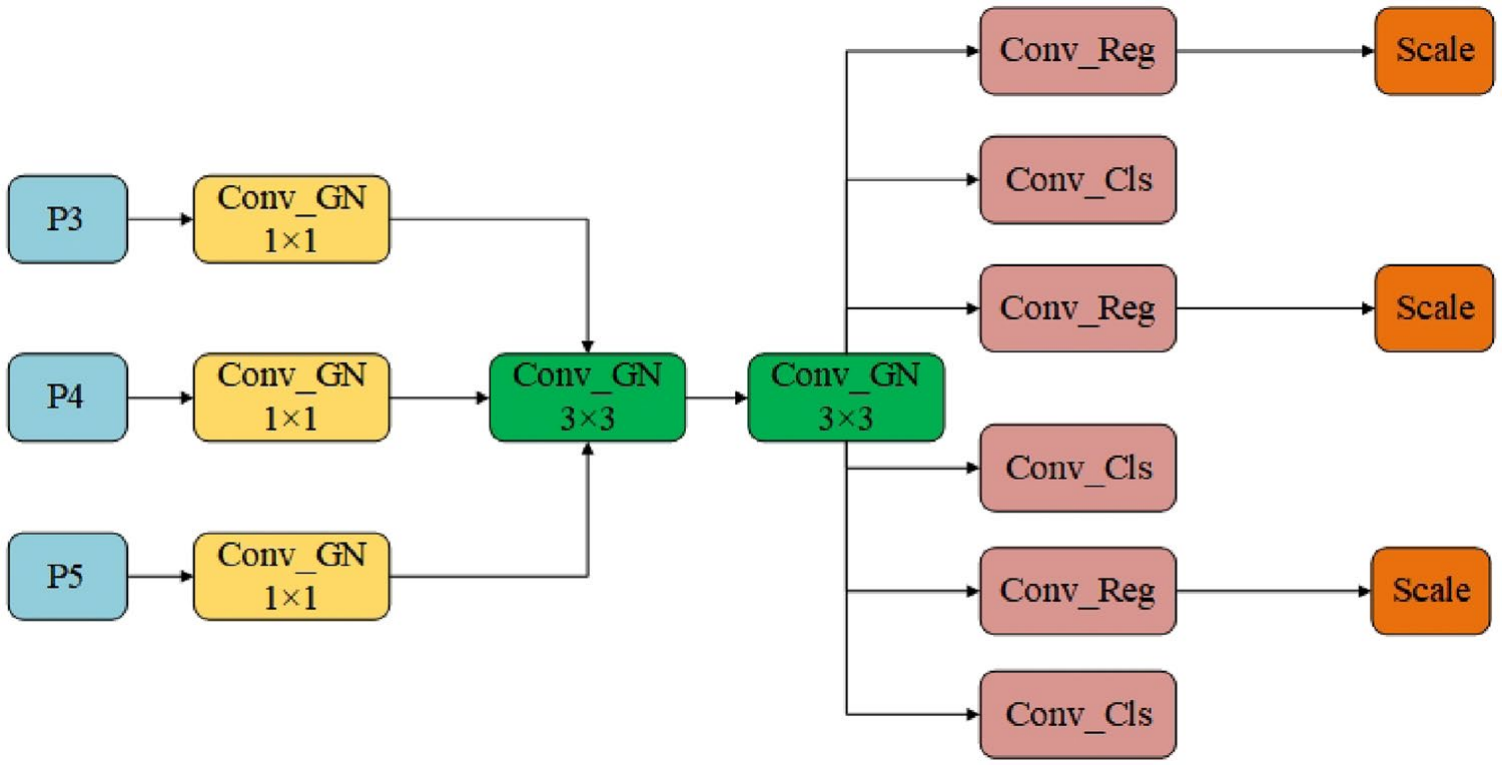
LSCD-Head module.

In order to solve the above two problems, we propose a new head structure, named LSCD-Head (Lightweight Shared Convolutional Detection Head). We introduce GroupNorm convolution in this head structure, which has been proved in FOCS papers^45^ to greatly enhance the localization and classification performance. The structure is shown in Fig. 11.

The core idea of this structure design is to replace the two common convolutions used by the three heads with a shared GroupNorm convolution ( as shown in the green and yellow parts of Fig. 11 ). At the same time, in order to deal with the problem that the target scale detected by each detection head is inconsistent, the scale layer is used to scale the features. Through the above structure, we can effectively reduce the number of parameters while allowing the detection head to have higher multi-scale sensing capabilities for deployment on resource-constrained devices.

## Datasets and evaluation parameters

### RDD2022

This paper utilizes the open-source RDD 2022 dataset^46^ for model training. RDD 2022, released by the University of Tokyo, consists of 47,420 road images from six countries: Japan, India, Czech Republic, Norway, the United States and China. These images have been annotated with over 55,000 road damage incidents. The dataset captures four types of road damage: D00 (longitudinal cracks), D10 (transverse cracks), D20 (alligator cracks), and D40 (potholes). In the experiment, 10,000 photos were randomly selected from the RDD 2022 dataset from the six countries. Then, 8000 photos were chose for training and 2000 photos for validation. The ratio of the training set to the validation set is 8:2.

### Evaluation parameters

The development language of this model is mainly Python, using the open-source deep learning framework PyTorch as the network framework, and accelerating training using CUDA 11.8. The hardware testing environment of this model includes an Intel(R) Core(TM) i7-10750H CPU and an NVIDIA RTX 2060 GPU with 6GB of memory. During training, the input images are set to 640 $\times$ 640, and SGD is used as the optimization function for model training. The model training epoch is set to 300, with a batch size of 16, and an initial learning rate of 0.01.

The evaluation metrics used in this paper include F1 score, mean Average Precision (mAP), number of parameters (Params), and Giga Floating Point Operations per Second (GFLOPs). Among these, precision and recall are used as basic metrics, with F1 score and mAP calculated serving as the final evaluation metrics to measure the model’s recognition accuracy. The subsequent equations can be utilized to compute these metrics.

6 $$\begin{aligned} \text {Precision}= & {} \frac{\textrm{TP}}{\mathrm {TP+FP}} \end{aligned}$$

7 $$\begin{aligned} \text {Recall}= & {} \frac{\textrm{TP}}{\mathrm {TP+FN}} \end{aligned}$$

8 $$\begin{aligned} \textrm{mAP}= & {} \frac{1}{n} \sum _{i=1}^{n} \int _{0}^{1} {\text {Precison}}(\text{ Recall}) d(\text {Recall}) \end{aligned}$$

9 $$\begin{aligned} \textrm{F1}= & {} 2 \frac{\text {Precision} \times \text {Recall}}{\text {Precision}+ \text {Recall}} \end{aligned}$$

In the aforementioned formulas, the meanings of each variable are as follows:

Precision refers to the ratio of correctly predicted positive samples among all samples predicted as positive.

Recall is calculated based on the proportion of all targets correctly predicted.

TP represents the number of correct targets in the detection results, FP represents the number of incorrect targets in the detection results and FN represents the number of missing targets among the correct targets.

The mAP refers to the average accuracy of n categories.

The F1-score comprehensively considers precision and recall, reflecting the overall performance of the network more comprehensively.

## Model training

**Figure 12:**
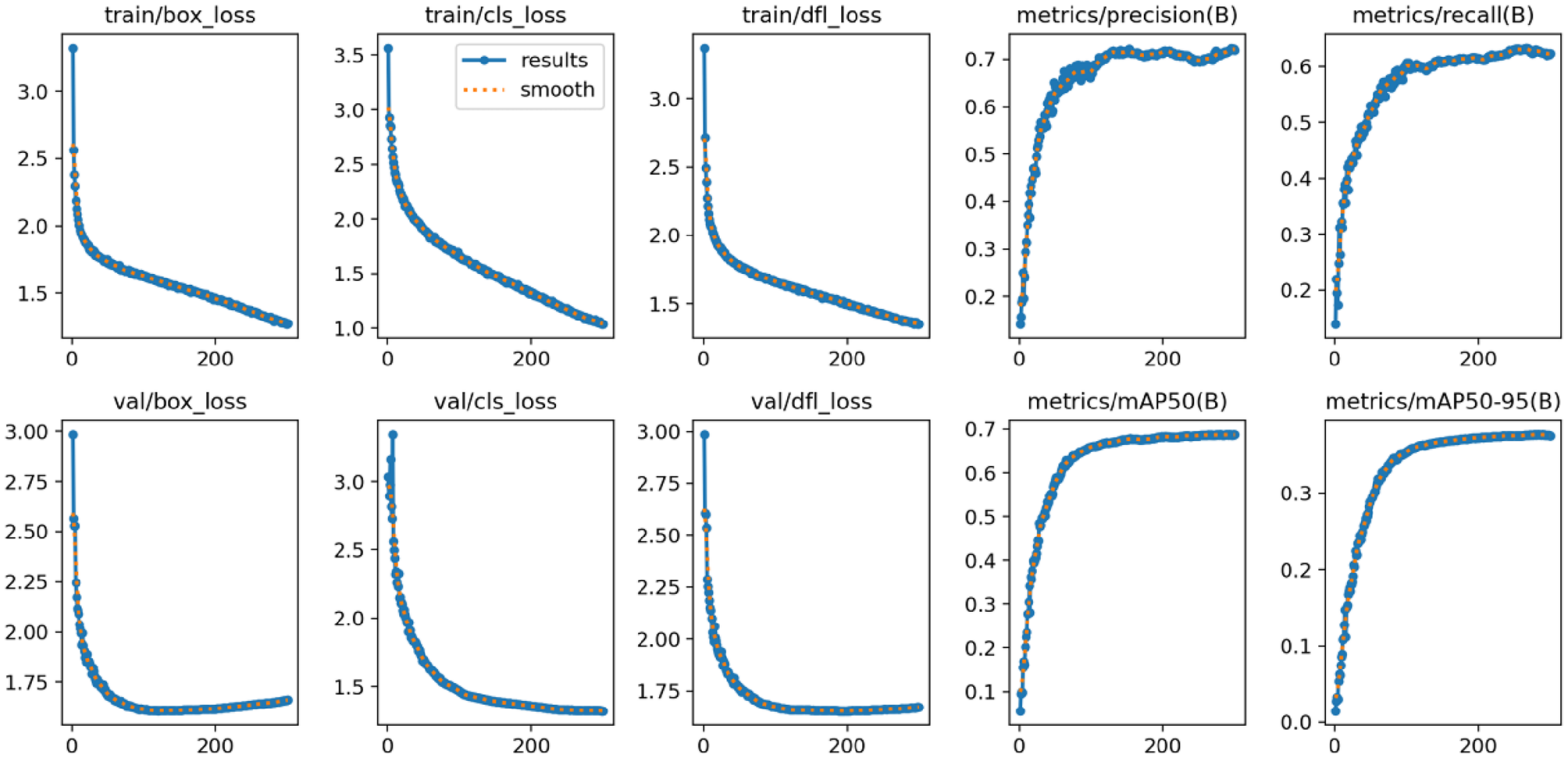
Training results of the proposed YOLOv8-PD.

**Figure 13:**
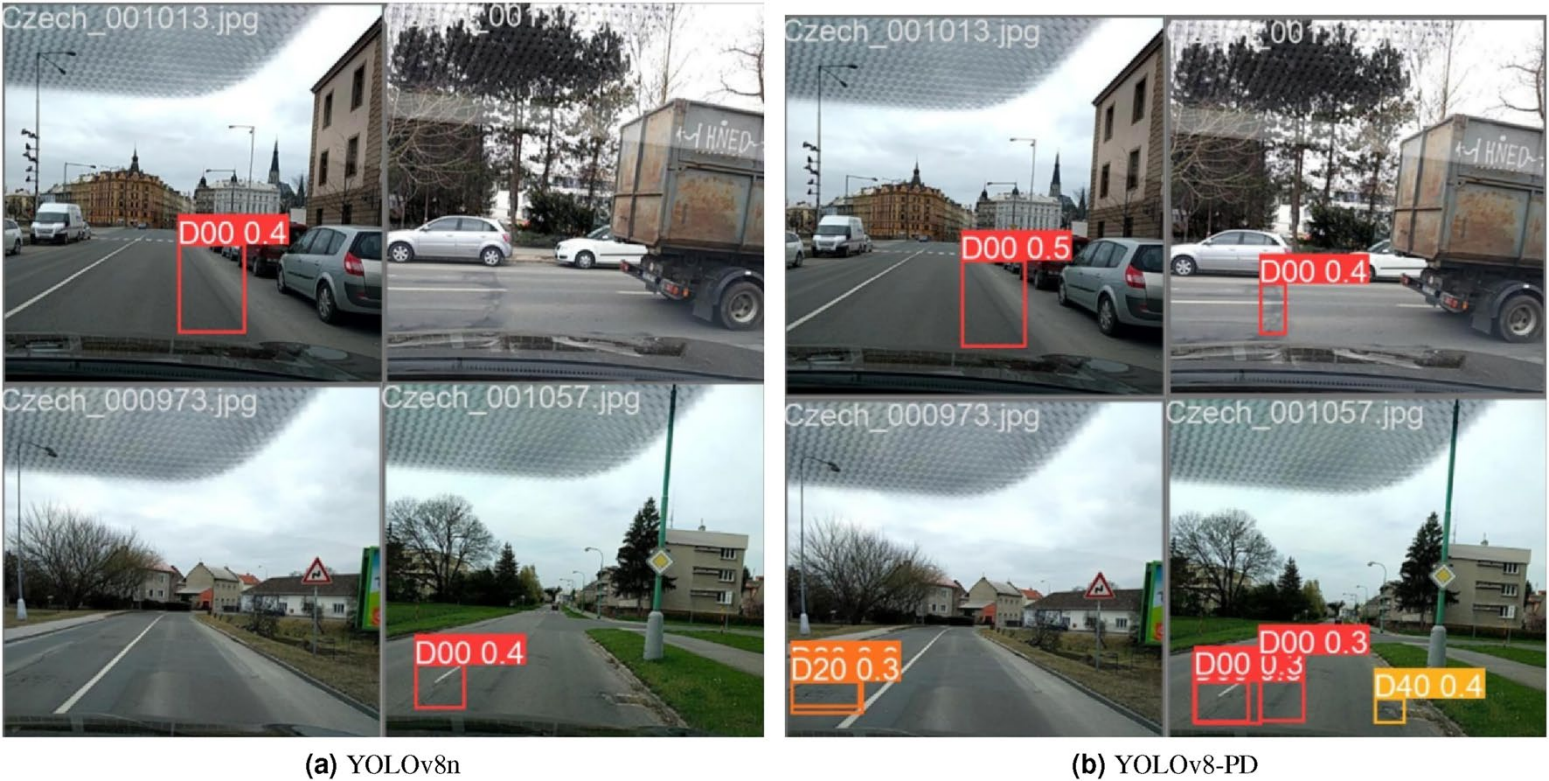
The detection effect of YOLOv8-PD and YOLOv8n.

When training the network model for road damage detection, the dimensions of input images were uniformly adjusted to 640 $\times$ 640 $\times$ 3. The SGD optimizer was utilized for a total of 300 epochs. Additionally, to enhance detection capabilities, Mosaic data augmentation technique was employed in the last 10 training epochs. This adjustment aims to improve the model’s robust performance in detecting road damage, as depicted in Fig. 12, illustrating the training results. The effectiveness of the algorithm was also verified experimentally, as shown in Fig. 13, demonstrating the detection performance.

## Results

### Ablation experiment

In order to investigate whether the improvement modules of YOLOv8-PD are effective, this paper conducted ablation experiments on the RDD2022 dataset, and the experimental results are shown in Table 1. The improved algorithm utilizes a more efficient network structure to enhance the YOLOv8n architecture, thereby improving accuracy while reducing the model’s parameters and computational complexity. It also demonstrates that the C2fGhost module does not reduce the algorithm’s accuracy, but reduces the model’s parameters and computational complexity. The introduction of the LSKA attention mechanism only increases a small number of parameters but effectively improves detection accuracy. Combining the above improvements with the YOLOv8n algorithm minimizes the model size, with the model parameters reduced to only 2.3M and computational complexity to only 6.2G, reducing by 27.6% and 25%, respectively. This effectively reduces the difficulty and cost of deploying the model on mobile terminals while significantly improving accuracy to meet real-time requirements.

**Table 1:** Ablation experiment on each improved module.

C2fGhost	LSCD-Head	LSKA	BOT	mAP50	mAP50:95	F1-score	Params/M	GFLOPs
				0.692	0.38	0.67	3.1	8.2
$\checkmark$				0.694	0.39	0.67	2.6	7.2
	$\checkmark$			0.705	0.385	0.68	2.36	6.5
		$\checkmark$		0.695	0.391	0.67	3.17	8.4
			$\checkmark$	0.701	0.39	0.68	3.25	8.5
	$\checkmark$		$\checkmark$	0.693	0.38	0.67	2.58	6.7
	$\checkmark$	$\checkmark$	$\checkmark$	0.702	0.391	0.68	2.13	5.8
$\checkmark$	$\checkmark$	$\checkmark$	$\checkmark$	0.706	0.395	0.68	2.3	6.1

### Comparison experiment on attention mechanisms

The experiment also utilized other attention mechanism modules, such as EMA attention^47^ and CA attention^48^. The experimental results are shown in Table 2. From Table 2, it can be seen that compared to other attention mechanism modules, the LSKA module has the highest detection accuracy, with a 0.9% increase in mAP50.

**Table 2:** Comparison experiment on attention mechanisms.

YOLOv8n	mAP50	mAP50:95	F1-score	Params/M	GFLOPs
+CBAM	0.686	0.376	0.66	3.35	9.1
+CA	0.694	0.391	0.67	3.03	8.1
+EMA	0.69	0.389	0.67	3.04	8.5
+LSKA (ours)	0.695	0.391	0.67	3.17	8.4

### Comparison experiment on detect head

The LSCD-Head, designed by combining the advantages of GroupNorm and shared convolution, is more lightweight and retains the advantages of detecting small objects. Table 3 presents a performance comparison experiment among LSCD-head, Efficient-Head, and Seam-Head. It can be observed that the detection accuracy is highest when using the LSCD-head module, which is also lighter, with a 1.03% increase in mAP50, and a decrease in the number of parameters and computational cost by 31.36% and 26.15%, respectively.

**Table 3:** Comparison experiment on detect head.

YOLOv8n	mAP50	mAP50:95	F1-score	Params/M	GFLOPs
+Efficient-Head	0.699	0.386	0.67	3.86	8.2
+SEAM-Head	0.701	0.386	0.68	2.91	7.4
+MultiSEAM-Head	0.695	0.386	0.68	4.92	7.4
+LSCD-Head (ours)	0.705	0.385	0.68	2.36	6.5

### Comparison experiment on different convolutions in neck

The C2fGhost module, designed to leverage the advantages of GhostNet and C2f. convolutions, is lighter while maintaining the accuracy of the original convolution. Table 4 presents a performance comparison experiment among C2fGhost, C2fGhostDynamicConv, and C2fReVitBlock, revealing that the detection accuracy is highest when using the C2fGhost module, with mAP50 increasing by 0.4 percentage points.

**Table 4:** Comparison experiment on different convolutions in neck.

YOLOv8n	mAP50	mAP50:95	F1-score	Params/M	GFLOPs
+C2fGhostDynamicConv	0.69	0.386	0.68	2.17	6
+C2fReVitBlock	0.692	0.389	0.68	2.4	6.3
+C2fGhost (ours)	0.694	0.395	0.68	2.3	6.1

### Comparison experiment of different models

The ablation experiment results have confirmed the effectiveness of YOLOv8-PD. Furthermore, This paper compares with other algorithms: Faster-RCNN, Cascade-RCNN, YOLOv3-tiny, YOLOv4-tiny, YOLOv5n, YOLOv5s, YOLOv5l, YOLOv7, YOLOv8n and YOLOv8-RD^49^. The YOLOv8-RD algorithm is a lightweight road damage detection algorithm proposed by Song Li. It is one of the advanced papers on road defect detection in recent years. The experiment was carried out under the same experimental conditions as the YOLOv5 model improved by Guo^50^ and the YOLOv7 model improved by Pham V^51^. Compared to the aforementioned algorithms, YOLOv8-PD achieves the best performance in terms of mAP50, mAP50:95, and F1-Score. Considering both accuracy and speed metrics, the proposed algorithm balances detection accuracy and real-time performance, performing better in road defect detection tasks. The comparison results are shown in Table 5.

**Table 5:** Comparison of evaluation index among diferent models.

Algorithms	mAP50	mAP50:95	F1-score	Params/M	GFLOPs
Faster-RCNN	0.512	0.225	0.494	137.1	370.2
Cascade-RCNN	0.548	0.25	0.564	81.9	110.6
YOLOv3-tiny	0.58	0.225	0.57	8.67	12.9
YOLOv4-tiny	0.427	0.159	0.39	6.1	9.8
YOLOv5n	0.595	0.254	0.6	1.7	4.2
YOLOv5s	0.672	0.355	0.67	7.03	16
YOLOv5l	0.647	0.305	0.64	64.1	108.3
YOLOv7-tiny	0.648	0.312	0.64	6.02	13.2
YOLOv8n	0.692	0.38	0.67	3.1	8.2
YOLOv8-RD	0.693	0.386	0.66	2.56	7.2
YOLOv8-PD (ours)	0.706	0.395	0.68	2.3	6.1

### Generalization experiment

To evaluate the generalization capability of the YOLOv8-PD model, this paper employs the publicly available RoadDamage dataset, which consists of 3321 actual road damage images captured using smartphone cameras, with a resolution of approximately 1080P. Similar to previous experimental designs, road damage targets are categorized into four classes: D00 (longitudinal cracks), D10 (transverse cracks), D20 (alligator cracks), and D40 (potholes), with a training-to-validation ratio of 8:2. The generalization experiment results on this dataset, as shown in Table 6, indicate that the performance of YOLO-PD remains superior to YOLOv8n. Due to variations in target quantity and image quality across different datasets, the degree of improvement in evaluation metrics also varies. On the RoadDamage dataset, mAP50, mAP50:95 and F-Score see improvements of 4.1, 2.1 and 0.5 percentage points, respectively. Taken together, these results confirm the strong generalization capability of the proposed algorithm.

**Table 6:** Generalization experiment on RoadDamage dataset.

Dataset	Algorithms	mAP50	mAP50:95	F1-score
RoadDamage	YOLOv8n	0.601	0.272	0.58
	YOLOv8-PD	0.642	0.293	0.63
RDD2022	YOLOv8n	0.692	0.38	0.67
	YOLOv8-PD	0.706	0.395	0.68

### Comparative experiment on the detection effect of different categories of road damages

The RDD2022 dataset captures four types of road damage, namely D00 (longitudinal cracks), D10 (transverse cracks), D20 (alligator cracks) and D40 (potholes). Figure 13 shows the detection effect of YOLOv8-PD algorithm on RDD2022 dataset compared with YOLOv8n algorithm. The improved algorithm shows better results than the original algorithm in the following four scenarios. In the first image, the original algorithm of D20 target is incomplete, and the detection of the improved algorithm is more accurate. In the second graph, the improved algorithm detects a D00 target that the original algorithm does not detect. In the third image, the improved algorithm detects two D20 targets that the original algorithm does not detect. In the forth image, both algorithms identify the D00 target, but the improved algorithm detects the D40 target additionally.

**Figure 14:**
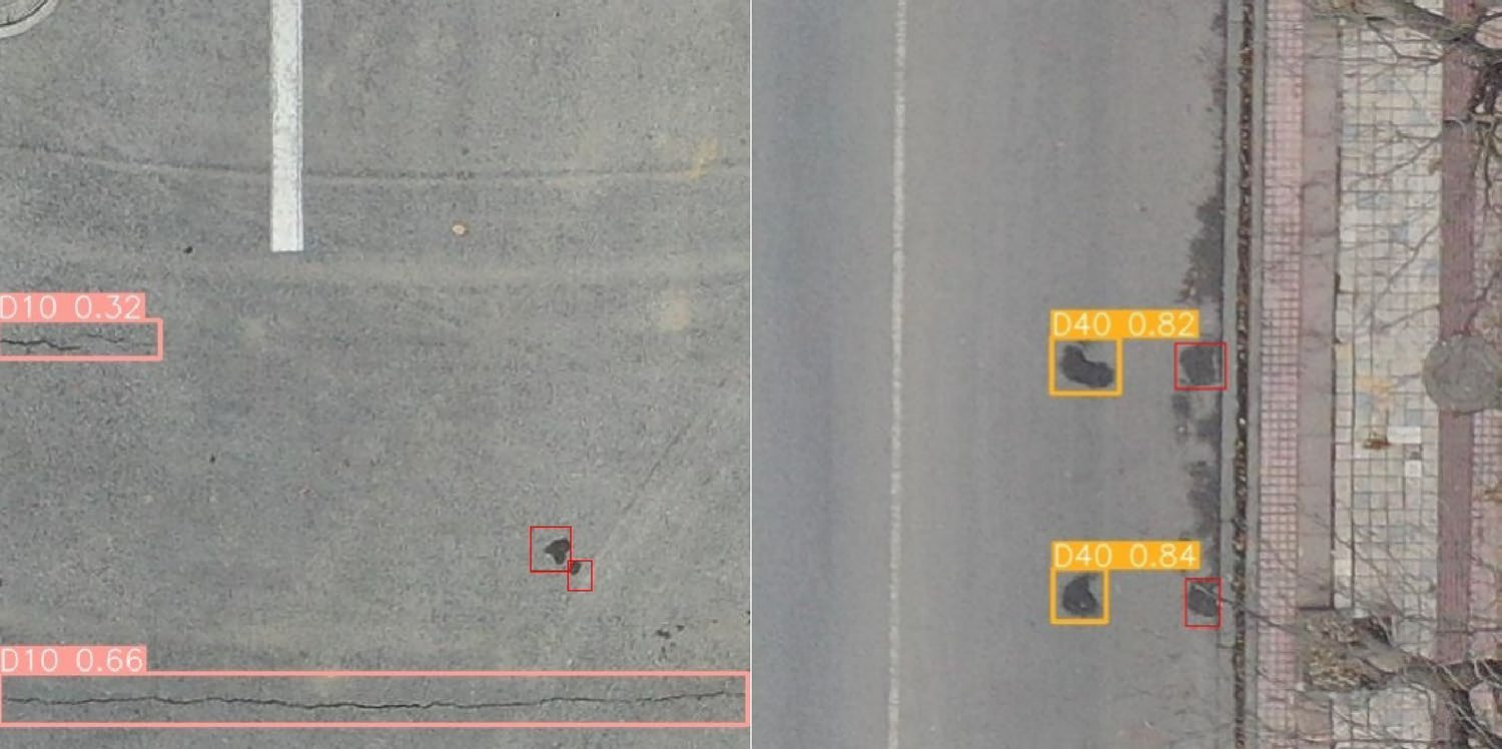
Undetected small potholes using YOLOv8-PD algorithm.

Figure 14 shows some failed test samples detected by YOLOv8-PD algorithm. D40 (potholes) targets were not detected in the two images displayed. This shows that the algorithm has the problem of missed detection in face of multi-hole scene and small hole scene. Because the algorithms for extracting crack features and extracting hole features are not well compatible, this provides an improved space for subsequent algorithms.

In order to further verify the detection effect of the model on different targets, Table 7 shows the performance of YOLOv8n and the improved model YOLOv8-PD under different damage conditions. The data show that the detection accuracy of YOLOv8-PD in four categories is higher than that of YOLOv8n, and the detection effect of category D00 (longitudinal cracks) is the most obvious. Compared with YOLOv8n, mAP50 and mAP50-90 increased by 2.0% and 1.1%, respectively. It should be noted that among all road crack targets, D40 (potholes) has the lowest detection accuracy. This is because the D40 target is tiny and the number of training samples is small, and the model is difficult to learn more features. These experimental results show that the YOLOv8-PD algorithm can effectively detect road damage targets and accurately identify their location and category, showing strong robustness and accuracy.

**Table 7:** Comparison of detection results for various types of damage in the RDD2022 dataset.

Algorithms	Type	mAP50 (%)	mAP50:95 (%)
YOLOv8	D00	74.2	37
	D10	79.3	39.5
	D20	71.9	35.9
	D40	51.2	25.6
	ALL	69.2	38
YOLOv8-PD	D00	76.2(+2.0)	38.1(+1.1)
	D10	79.7(+0.4)	39.8(+0.3)
	D20	73.3(+1.4)	36.7(+0.7)
	D40	53.1(+1.9)	26.5(+0.9)
	ALL	70.6(+1.4)	39.5(+1.5)

## Discussion

Object detection algorithms using deep learning have proven to be effective in achieving high accuracy in a variety of tasks, making them a popular choice for machine learning practitioners. However, the complexity and computational requirements of incredible deep networks can make it challenging to deploy them in real-world applications where resources may be limited or the need for quick decision-making is crucial. Given this, it’s important to consider deep learning models using fewer trainable parameters that may not have the same level of accuracy but are more practical for deployment in the real world. While deeper networks still hold potential for improving accuracy, it may be necessary to balance that with considerations of practicality and feasibility in road damage detection tasks.

Pavement distress detection plays a crucial role in road protection. In this study, we present an efficient and lightweight YOLOv8-PD model designed specifcally for the detection of pavement distress. To decrease the size of the model, we introduce the C2fGhost module and the LSCD-Head detection head. Furthermore, the BOT transformer structure is introduced to boost accuracy in detecting long-range cracks. Additionally, the introduction of the LSKA mechanism comprehensively extracts local crack feature information in complex road environment.

The experimental results of the proposed YOLOv8-PD model on RDD2022 dataset for pavement distress detection indicate advantages compared to some current mainstream object detection and lightweight methods. It excels in evaluation metrics such as Precision, Recall, mAP, Parameters, Model size, and FPS. Although the accuracy of this algorithm in detecting pothole is only 53.1% and missing detection of D40 targets occured occasionally.The proposed approach still achieves an mAP of 70.6% and a speed of 111.9 frames per second, demonstrating its competence in pavement distress detection tasks.

## Conclusion

This paper proposes an improved road damage detection algorithm based on YOLOv8n, addressing the challenges faced by traditional YOLOv8n in object detection applications. In the proposed method, firstly, to enhance road crack detection accuracy, we use the BOT transformer structure. Then we use the LSKA Attention module to optimize the network to improve the model detection accuracy. Thirdly, the C2fGhost block is constructed in the neck network of YOLOv8-PD, strengthening the ability of feature extraction while simultaneously reducing computational load. Finally, a lightweight detection head module, LSCD-Head, is proposed to reduces the size of the model while maintaining detection accuracy and speed.

Experiments show that YOLOv8-PD has advantages such as low computational load and higher detection accuracy, meeting real-time requirements. Compared with existing models, this method achieves higher detection accuracy while reducing requirements for platform computing and storage capacity, making it easy to deploy on resource-constrained devices. Future research will focus on deploying the improved model on resource-constrained embedded detection devices and refining the proposed algorithm for practical applications.

## Data Availability

The data utilized in this paper is obtained through self-gathering and is made publicly available to make the study reproducible. It can be accessed at https://github.com/sekilab/RoadDamageDetector. If you want to request the complete dataset and code, please email the corresponding author.
